# Eurofever-lesons from last year

**DOI:** 10.1186/1546-0096-12-S1-I32

**Published:** 2014-09-17

**Authors:** Marco Gattorno

**Affiliations:** 1 UO Pediatria II, G. Gaslini Institute, Genoa, Italy

## 

Autoinflammatory diseases are rare disorders secondary to mutation of genes involved in the regulation of innate immunity. The main limitation to a better knowledge of Autoinflammatory diseases is related to the extreme fragmentation of the diagnosed cases that are spread over different centers and countries. The general aim of the Eurofever Project was to build an international registry on Autoinflammatory diseases.

A web-based registry collecting baseline and cross-sectional clinical information on Autoinflammatory diseases is available in the member area of the PRINTO web-site (http://www.printo.it). The registry is open to all pediatric and adult Centers with a specific interest in Autoinflammatory diseases. The following monogenic autoinflammatory diseases were considered: Familial Mediterranean Fever (FMF), Cryopyrin-associated periodic syndromes (CAPS), TNF receptor-associated periodic syndrome (TRAPS), mevalonate kinase deficiency (MKD), Blau syndrome, pyogenic arthritis, pioderma and acne (PAPA) syndrome, deficiency of IL-1 receptor antagonist (DIRA), NLRP12-mediated periodic fever. Information on CRMO, Behçet’s disease, PFAPA and undefined periodic fevers were also collected.

2916 patients, from 95 centers in 53 countries, have been enrolled in the registry during the first 36 months. Baseline demographic data (country of residence, disease onset, disease duration, mutations, family history ect) from all patients are now available. In 2275 (81%) complete information on clinical manifestations and responses to treatments is also available. The disease distribution of enrolled patients is: FMF 787 (621 with complete clinical data); TRAPS 237 (211 with complete clinical data); CAPS 207 (186 with complete clinical data); MKD 153 (133 with complete clinical data); Blau syndrome 62 (21 with complete clinical data); PAPA 19 (18 with complete clinical data); NLRP-12 mediated periodic fever 8 (6 with complete clinical data); DIRA and Majeed 3 and 2 patients, respectively (all with complete clinical data).

Among multifactorial autoinflammatory diseases: PFAPA 564 (402 with complete clinical data); CRMO 392 (370 with complete clinical data); pediatric Behçet disease 84 (68 with complete clinical data) and 205 patients with undefined periodic fever (174 with complete clinical data).

So far 8 papers involving 56 different authors and 32 centers have been published in high-rank international journals and other papers are in preparation.

A large registry of patients with Autoinflammatory diseases is available and, despite the expiring of the initial grant, the enrolment is still ongoing with an increasing number of centers involved. Eurofever represents a good example of how a disease-oriented registry can provide relevant scientific answers to many unknown clinical aspects of ultra-rare diseases. This aspects was the main reason of the relevant success of the enrolment we have observed.

## Disclosure of interest

None declared.

